# Heart Transplantation of the Elderly—Old Donors for Old Recipients: Can We Still Achieve Acceptable Results?

**DOI:** 10.3390/jcm11040929

**Published:** 2022-02-10

**Authors:** Moritz Benjamin Immohr, Hug Aubin, Ralf Westenfeld, Sophiko Erbel-Khurtsidze, Igor Tudorache, Payam Akhyari, Artur Lichtenberg, Udo Boeken

**Affiliations:** 1 Department of Cardiac Surgery, Medical Faculty and University Hospital Düsseldorf, Heinrich-Heine-University Düsseldorf, 40225 Düsseldorf, Germany; Moritz.Immohr@med.uni-duesseldorf.de (M.B.I.); Hug.Aubin@med.uni-duesseldorf.de (H.A.); Sophiko.Erbel-Khurtsidze@med.uni-duesseldorf.de (S.E.-K.); Igor.Tudorache@med.uni-duesseldorf.de (I.T.); Payam.Akhyari@med.uni-duesseldorf.de (P.A.); Artur.Lichtenberg@med.uni-duesseldorf.de (A.L.); 2 Division of Cardiology, Medical Faculty and University Hospital Düsseldorf, Heinrich-Heine-University Düsseldorf, 40225 Düsseldorf, Germany; Ralf.Westenfeld@med.uni-duesseldorf.de

**Keywords:** heart transplantation, age, elderly, frailty, demographic change

## Abstract

As society is ageing, an increasing prevalence of elderly heart failure patients will be expected. In order to increase the donor pool, acceptance of older donors might be a reasonable choice. All patients undergoing heart transplantation between 2010 and 2021 at a single department were retrospectively reviewed and divided into different study groups with regard to recipient (≤60 years (R^Y^) or >60 years (R^O^)) and donor age (≤50 years (D^Y^) or >50 years (D^O^). A total of *n* = 201 patients were included (D^Y^/R^Y^, *n* = 91; D^O^/R^Y^, *n* = 38; D^Y^/R^O^, *n* = 41; D^O^/R^O^, *n* = 31). Neither incidence of severe primary graft dysfunction (*p* = 0.64) nor adverse events, such as kidney failure (*p* = 0.27), neurological complications (*p* = 0.63), infections (*p* = 0.21) or acute graft rejection (*p* = 1.00), differed between the groups. However, one-year survival was impaired in the D^O^/R^O^ group (56.0%) compared to the other groups (D^Y^/R^Y^: 86.1%, D^Y^/R^O^: 78.8%, D^O^/R^Y^: 74.2%, *p* = 0.02). Given the impaired one-year survival, acceptance of grafts from old donors for old recipients should be performed with caution and by experienced centres only. Nevertheless, because of the otherwise dismal prognosis of elderly heart failure patients, transplantation of patients may still improve the therapy outcome.

## 1. Introduction

Congestive heart failure is a global burden of disease affecting millions of people worldwide [1,2,3]. Among adults and the elderly, it is one of the leading causes for hospitalisation and origin of tremendous costs for health care systems [1,2,3]. Due to current demographic changes, numbers of heart failure patients are expected to further increase within the next decades [2]. By now, heart transplantation (HTx) is the standard of care for end-stage heart failure [4,5]. However, especially elderly heart failure patients often suffer from a variety of concomitant diseases and frailty, which has been reported to presently affect 45% of heart failure patients [6]. Transplanting these elderly and frail patients might be challenging [7]. To expand the donor pool for this increasing number of older patients on the transplant waiting list, accepting more and more old and marginal donors might be an option as it has been successfully performed for other donor organs [7,8,9]. However, cardiac grafts of old donors carry a risk for impaired long-term survival [10,11].

In order to investigate possible effects of donor and recipient age matching for the outcome after HTx, we aimed to analyse the postoperative outcome for young and old recipients of cardiac grafts from young and old donors. We therefore retrospectively reviewed our institutional data of the last decade and compared the outcome after HTx for different groups of donor and recipient age matching.

## 2. Materials and Methods

### 2.1. Patients and Study Design

**Figure 1:**
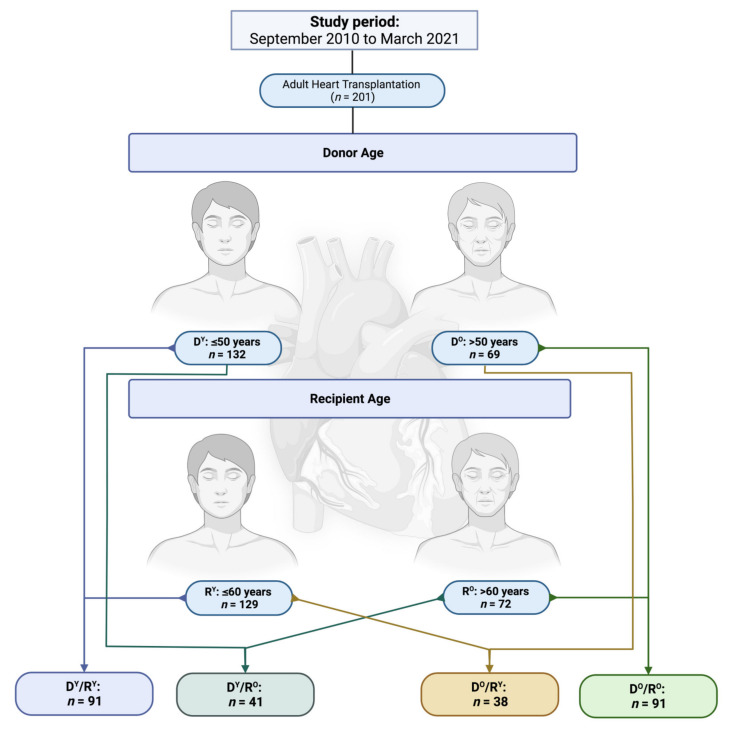
Study groups.

All adult patients (*n* = 201) who underwent HTx between September 2010 and March 2021 in our department were prospectively enrolled in an institutional database. Patients were retrospectively reviewed and those who underwent cardiac re-transplantation were excluded. Afterwards, patients were divided into four study groups with regard to the recipient and donor age matching (Figure 1). Recipients aged 60 years or younger (*n* = 129) were declared as young recipients (R^Y^). Correspondingly, recipients undergoing HTx over 60 years of age were declared as old recipients (R^O^, *n* = 72). In line with the current literature [10,11], for donors, age limit was set at 50 years (donor age ≤ 50 years: young donors (D^Y^, *n* = 132), donor age > 50 years: old donors (D^O^, *n* = 69)). Accordingly, young recipients with young donors (D^Y^/R^Y^, *n* = 91) were compared to young recipients with old donors (D^O^/R^Y^, *n* = 38) as well as old recipients with young donors (D^Y^/R^O^, *n* = 41) and old recipients with old donors (D^O^/R^O^, *n* = 31).

### 2.2. Study Objectives and Follow-Up Period

Relevant donor and recipient parameters were examined and impact of donor and recipient age matching on the postoperative morbidity and mortality was analysed. Patients were postoperatively followed-up every three to six months on a regular basis. Postoperative one-year survival was defined as the primary endpoint and impaired postoperative survival was hypothesised for old recipients with old donor organs. In addition, perioperative adverse events, such as acute kidney failure, neurological complications or bleeding complications, were defined as secondary endpoints of the study.

### 2.3. Surgical Procedure and Perioperative Management

HTx was performed with either orthotopic bicaval or biatrial technique. For immunosuppression a standardised institutional protocol consisting of tacrolimus, mycophenolate mofetil and prednisolone. Potential graft rejection was routinely examined by right ventricular endomyocardial biopsies and addressed with high-dose prednisolone therapy for at least three consecutive days. In case of antibody-mediated rejection, therapy was amended by immunoabsorption or plasmapheresis, anti-T-lymphocyte IgG and intravenous IgM-enriched human immunoglobulin. Patients suffering from primary graft dysfunction were treated following an institutional standard operating procedure covering adequate catecholamine therapy with epinephrine and norepinephrine and a relatively liberal regime of early implantation of veno-arterial extracorporeal membrane oxygenation (va-ECMO) and percutaneous microaxial pumps (Impella 5.0, Abiomed, Inc., Danvers, MA, USA).

### 2.4. Statistics

For statistical analyses SPSS Statistics 26 (IBM Corporation, Armonk, NY, USA) was used. All results are displayed as mean values with the standard deviation (SD) respectively percentage of the whole. Because of the small and unbalanced groups sizes, Gaussian distribution was not assumed, and variables were therefore compared by either non-parametric two-tailed Kruskal–Wallis tests or Fisher–Freeman–Halton tests. In case of statistically significant results (*p* < 0.05), additional post-hoc analyses were used by a Bonferroni correction. Postoperative survival after HTx was calculated by the Kaplan–Meier method and compared by log-rank test. Detailed information of the post-hoc tests are displayed in the Supplementary Table S1.

## 3. Results

### 3.1. Pre-Transplant Recipient Parameters

Detailed preoperative recipient parameters are displayed in Table 1. As given by the study protocol, there was a significant difference between the four groups with regard to the recipient age with a mean age of 48 years for D^Y^/R^Y^ patients and 65 years in the D^O^/R^O^ group. Recipient age ranged from 22 years (D^Y^/R^Y^) to 73 years (D^O^/R^O^). Younger recipients were much more often transplanted with high urgency wait list status compared to old recipients (*p* < 0.01). This was also underlined by the increased incidence of pre-transplant mechanical ventilation in the D^Y^/R^Y^ and D^O^/R^Y^ group compared to the other two groups. The same effect was also numerically observed for pre-transplant cardiopulmonary resuscitation. Interestingly, we did not observe any other differences with regard to the incidence of preoperative risk factors for impaired outcome or concomitant diseases. Especially, there was no difference in the incidence of previous mechanical circulatory support (ventricular assist devices or extracorporeal life support).

### 3.2. Pre-Transplant Donor Parameters

Detailed preoperative donor parameters are displayed in Table 2. Differences in demographic data of the four groups are once again given by the study protocol. Minimum donor age was 15 years (D^Y^/R^Y^) and maximum was 67 years (D^O^/R^O^). Although donor sex distribution and body mass index were inhomogeneous between the four groups, predicted heart mass ratio of the recipients and donors was comparable, indicating no relevant differences regarding organ size mismatch. Younger donors were much more likely to be resuscitated before recovery of the organs. Nevertheless, there were no differences regarding catecholamine therapy and concomitant diseases, indicating a similar distribution rate of marginal donors between the four groups.

### 3.3. Operative Outcome

Table 3 shows the postoperative outcome of the patients. While warm ischemia did not differ between the groups, the average transport time was slightly prolonged in the groups with the younger donors compared to the corresponding groups of similar recipient age (*p* = 0.02). Consequently, total graft ischemic time was also slightly prolonged. There was a strong trend towards increased postoperative epinephrine doses in the D^O^/R^O^ group with about 50% higher peak concentration compared to the D^Y^/R^Y^ group (*p* = 0.05). Nevertheless, incidence of va-ECMO implantation and postoperative support duration was comparable between all groups. Perioperative severe adverse events were also comparable between all groups with no advantages for any of the four groups. In line with these results, duration of postoperative mechanical ventilation and hospital stay also did not differ.

### 3.4. Postoperative Survival

**Figure 2:**
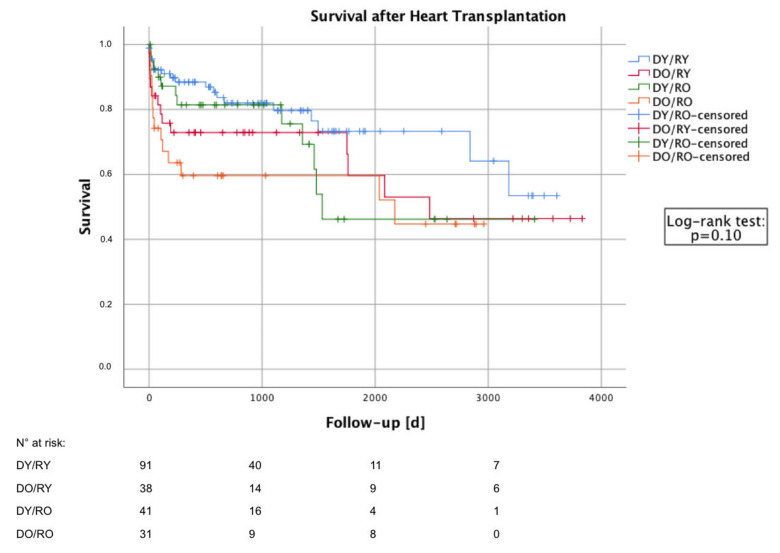
Estimated longer-term survival after heart transplantation by Kaplan–Meier method. Patients were divided into four study groups with regard to the donor and recipient age (donor age ≤ 50 years and recipient age ≤ 60 years: D^Y^/R^Y^, *n* = 91; donor age > 50 years and recipient age ≤ 60 years: D^O^/R^Y^, *n* = 38; donor age ≤ 50 years and recipient age > 60 years: D^Y^/R^O^, *n* = 41; donor age > 50 years and recipient age > 60 years: D^O^/R^O^, *n* = 31).

Mean postoperative follow-up was about three years (991 days, SD: 1012 days) with a maximum of ten and a half years (3831 days). As shown in Table 3, 30-day survival was best for recipients of grafts from young donors (D^Y^/R^Y^ = 94.4% and D^Y^/R^O^ = 95.0% compared to D^O^/R^Y^ = 84.2% and D^O^/R^O^ = 80.6%, *p* = 0.05). The primary end-point of one-year survival was still best for D^Y^/R^Y^ (86.1%), followed by comparable results for D^Y^/R^O^ (78.8%) and D^O^/R^Y^ (74.2%) but deeply impaired for D^O^/R^O^ (56.0%) (*p* = 0.02). The cause of death within the first 30 days as well between 30 days and 1 year did not differ between the four groups. Within the first 30 days, multiple causes of death appeared; however, after 30 days, infective complications were the leading cause of death. Six patients died because of graft failure: three grafts from young and three grafts from old donors. In addition, the Kaplan–Meier survival curve is shown in Figure 2. Log-rank test (*p* = 0.10) identified no statistical significance between the four curves, but numerical differences indicated similar mid- to long-term results to those for short-term survival.

## 4. Discussion

In the coming years, there may be a rise in elderly end-stage heart failure patients due to a continuing demographic change leading to an ageing society. As HTx remains the gold standard of care, this rise will most likely also enter the transplant waiting list. In order to examine whether acceptance of older donors might be an option for those patients, we retrospectively analysed all of our transplant data from the last decade. Although we did not observe an increase in perioperative adverse events in the group of old recipients of organs from old donors, their postoperative survival was significantly impaired.

Except age, baseline characteristics of both the recipients as well as the donors were comparable between the groups. Therefore, the question arises as to why one-year survival of D^O^/R^O^ was only 56%. Donor age is a known risk factor for impaired post-transplant long-term survival [10,11]. However, we already observed this for the very short-term survival. In addition, donor age is also a strong and independent risk factor for primary graft dysfunction, which we did not observe [12,13,14,15].

It was no surprise that patients of the D^Y^/R^Y^ group had the best outcome as this has been reported in several previous studies [14,15,16,17]. In order to interpret our data of the D^O^/R^O^ group, it is important to review the results of the D^Y^/R^O^ and D^O^/R^Y^ patients who had similar short-term survival. First, D^Y^/R^Y^ patients had a better outcome than D^O^/R^Y^ as well as D^Y^/R^O^ patients. Secondly, D^O^/R^Y^ patients had comparable outcome to D^Y^/R^O^ patients. Finally, D^Y^/R^O^ patients are superior to the D^O^/R^O^ group. Similar results have recently been described in an Italian single-centre retrospective analysis as well as a retrospective review of the United Network for Organ Sharing (UNOS) registry [14,15]. Nevertheless, the implications of these results represent some kind of ethical dilemma. First, young donors should be allocated to every recipient, as this was best for all recipient ages. However, due to a continuous decline in organ donation, there is a lack of suitable donor organs in the Euro transplant region today [18,19]. Although D^O^/R^Y^ were comparable to D^Y^/R^O^, allocating young donors primary to old recipients will still be questionable because donor age is a risk factor for impaired long-term outcome and these young recipients will then miss the even better outcome of the D^Y^/R^Y^ group [10,11,18,19]. 

Implantation of left ventricular assist devices (LVAD) has gained increasingly more popularity in the elderly [20]. Unfortunately, risk for perioperative morbidity and mortality is also significantly increased compared to younger patients with reported in-hospital mortality of up to 50% in patients of 65 years and older [20,21,22]. Therefore, this is also a unsatisfying alternative to HTx for elderly patients.

Age itself is a strong and independent risk factor for mortality of heart failure patients [23,24]. In a large meta-analysis, Jones and colleagues reported a five-year survival after first diagnosis of heart failure of less than 50% for patients aged ≥ 75 years compared to about 80% for those aged ≤ 65 years [24]. This prognosis may be further impaired by frailty and concomitant diseases [6]. Therefore, in order to solve the mentioned ethical dilemma of missing suitable cardiac grafts from young donors for both groups of young and old recipients, individual consensus decisions with all related medical professions and the patient seemed to be crucial. First, individually shared decisions as to whether an elderly patient should be enrolled to stay on the HTx waiting list should be made in relation to their individual health status (urgency, frailty, concomitant diseases, suitability for LVAD implantation, etc.) and the predicted post-transplant survival [25]. Afterwards, the best offered donor organ should be accepted for elderly patients as with every patient on the waiting list.

The scientific value of our data is limited by the study’s single-centre and retrospective design. The relatively small group sizes prohibited propensity score matching. In addition, the short follow-up period of the majority of patients combined with the known disproportionally high first-year mortality after HTx most likely underestimates the longer-term survival of the cohort assessed by the Kaplan–Meier method. The high number of censored patients led to a relatively small remaining follow-up cohort that may represent a bias for the longer-term follow-up. Furthermore, due to the retrospective character of the study, pretransplant frailty of the patients could unfortunately not be assessed.

## 5. Conclusions

Prevalence of heart failure will further increase within the next years due to an ageing society. Accordingly, an increasing number of elderly patients will enter the waiting list for heart transplantation. In order to increase the donor pool, accepting older donors can be performed without increasing the incidence of perioperative adverse events for both young and old recipients. However, donor age seems to be more important for the posttransplant survival than the recipient age. As we observed significantly impaired one-year survival for old recipients of grafts from old donors, organ acceptance should be performed with caution and by experienced centres only. However, given the otherwise often dismal prognosis of elderly and frail end-stage heart failure patients, transplantation of individual patients may still distinctly improve the therapy outcome of certain patients.

## Figures and Tables

**Table 1:** Preoperative recipient parameters.

	D^Y^/R^Y^	D^O^/R^Y^	D^Y^/R^O^	D^O^/R^O^	*p*-Value
**Recipient Variables**	**(*n* = 91)**	**(*n* = 38)**	**(*n* = 41)**	**(*n* = 31)**	
Age, y (SD)	48 (11)	52 (8)	64 (3)	65 (3)	<0.01
Female gender, *n* (%)	26 (28.6)	12 (31.6)	8 (19.5)	7 (22.6)	0.59
Height, cm (SD)	175 (8)	173 (11)	176 (7)	174 (7)	0.57
Weight, kg (SD)	78 (16)	75 (16)	79 (16)	79 (13)	0.52
Body mass index, kg/m^2^ (SD)	25.7 (4.9)	25.2 (5.0)	25.5 (4.0)	26.2 (3.8)	0.64
Panel-reactive antibodies, % (SD)	3.1 (14.5)	1.3 (6.6)	3.1 (19.1)	0.2 (0.9)	0.66
High urgency wait list status, *n* (%)	52 (57.1)	19 (50.0)	19 (46.3)	7 (22.6)	0.01
Aetiology					
Ischemic cardiomyopathy, *n* (%)	24 (26.4)	18 (47.4)	23 (56.1)	16 (51.6)	0.20
Dilated cardiomyopathy, *n* (%)	55 (60.4)	19 (50.0)	16 (39.0)	13 (41.9)	
Other, *n* (%)	12 (13.2)	1 (2.6)	2 (4.8)	2 (6.4)	
Ventricular assist device, *n* (%)	50 (54.9)	17 (44.7)	23 (56.1)	18 (58.1)	0.66
Extracorporeal life support, *n* (%)	6 (6.7)	2 (5.3)	1 (2.4)	0 (0.0)	0.57
Concomitant diseases					
Diabetes mellitus, *n* (%)	17 (8.7)	9 (23.7)	9 (22.0)	6 (19.4)	0.58
Haemodialyis, *n* (%)	7 (7.8)	1 (2.6)	1 (2.6)	1 (3.2)	0.65
Smoking, *n* (%)	21 (23.1)	7 (18.4)	8 (19.5)	8 (25.8)	0.74
Arterial hypertension, *n* (%)	50 (54.9)	25 (65.8)	26 (63.4)	17 (54.8)	0.72
Pulmonary hypertension, *n* (%)	8 (8.8)	5 (13.2)	2 (4.9)	4 (12.9)	0.52
COPD, *n* (%)	7 (7.7)	2 (5.3)	2 (4.9)	4 (12.9)	0.60
Cardiopulmonary resuscitation, *n* (%)	13 (14.3)	5 (13.2)	5 (12.2)	0 (0.0)	0.17
Mechanical ventilation, *n* (%)	8 (8.8)	5 (13.2)	1 (2.4)	0 (0.0)	0.01
Blood transfusion, *n* (%)	8 (8.8)	1 (2.6)	2 (4.9)	1 (3.2)	0.58
Laboratory values					
Hemoglobin, g/dL (SD)	11.6 (2.4)	11.5 (2.3)	12.4 (1.9)	12.6 (2.4)	0.05
Bilirubin, mg/dL (SD)	1.0 (1.2)	0.8 (0.9)	0.8 (0.6)	1.4 (0.4)	0.83
Creatinine, mg/dL (SD)	1.4 (1.3)	1.3 (0.5)	1.5 (0.7)	1.4 (0.4)	0.19
AST, U/L (SD)	49 (87)	41 (34)	29 (15)	30 (12)	0.46
Lactate dehydrogenase, U/L (SD)	413 (460)	288 (142)	279 (108)	285 (86)	0.87

Preoperative recipient parameters. Patients were divided into four study groups with regard to the donor and recipient age (donor age ≤ 50 years and recipient age ≤ 60 years: D^Y^/R^Y^, *n* = 91; donor age > 50 years and recipient age ≤ 60 years: D^O^/R^Y^, *n* = 38; donor age ≤ 50 years and recipient age > 60 years: D^Y^/R^O^, *n* = 41; donor age > 50 years and recipient age > 60 years: D^O^/R^O^, *n* = 31). Detailed results for post-hoc analysis are displayed in Supplementary Table S1. COPD, chronic obstructive pulmonary disease; AST, aspartate aminotransferase; SD, standard deviation.

**Table 2:** Donor parameters.

	D^Y^/R^Y^	D^O^/R^Y^	D^Y^/R^O^	D^O^/R^O^	*p*-Value
**Donor Variables**	**(*n* = 91)**	**(*n* = 38)**	**(*n* = 41)**	**(*n* = 31)**	
Age, y (SD)	35 (10)	56 (4)	38 (10)	58 (10)	<0.01
Female gender, *n* (%)	38 (41.8)	23 (60.5)	12 (29.3)	15 (48.4)	0.04
Height, cm (SD)	176 (9)	172 (8)	177 (6)	173 (8)	0.05
Weight, kg (SD)	80 (15)	79 (11)	79 (17)	81 (15)	0.81
Body mass index, kg/m^2^ (SD)	25.6 (4.1)	26.7 (3.1)	25.2 (5.0)	27.9 (7.0)	0.01
Predicted Heart Mass Ratio, % (SD)	13.8 (10.4)	14.7 (13.4)	12.6 (9.7)	11.6 (7.9)	0.87
Cardiopulmonary resuscitation, *n* (%)	24 (26.4)	3 (7.9)	19 (46.3)	6 (19.4)	<0.01
Duration, min (SD)	18 (13)	13 (3)	17 (13)	22 (17)	0.92
Norepinephrine, µg/kg/min (SD)	0.12 (0.16)	0.14 (0.33)	0.14 (0.21)	0.10 (0.09)	0.68
Ejection fraction, % (SD)	61 (9)	62 (10)	57 (10)	62 (7)	0.28
Concomitant diseases					
Arterial hypertension, *n* (%)	14/41 (34.1)	18/25 (72.0)	10/22 (45.5)	16/20 (22.2)	<0.01
Diabetes mellitus, *n* (%)	6/37 (16.2)	2/11 (18.2)	0/15 (0.0)	5/10 (50.0)	0.02
Smoking, *n* (%)	49/76 (64.5)	16/30 (53.3)	21/39 (53.8)	14/26 (53.8)	0.56
Drug abuse, *n* (%)	8/75 (10.7)	1/31 (3.2)	8/34 (23.5)	0/24 (0.0)	0.02
Laboratory values					
Hemoglobin, g/dL (SD)	10.1 (2.8)	9.9 (1.9)	10.3 (2.9)	10.3 (2.4)	0.90
White blood cells, 1 × 10^9^/L (SD)	15.1 (5.8)	14.9 (5.8)	14.3 (4.4)	21.0 (39.2)	0.89
Lactate dehydrogenase, U/L (SD)	510 (681)	352 (257)	525 (414)	347 (191)	0.04
Creatinine kinase, U/L (SD)	2029 (8139)	438 (643)	1068 (2326)	682 (1350)	0.11
C-reactive protein, mg/L (SD)	163 (232)	234 (416)	157 (110)	151 (96)	0.55

Donor parameters. Patients were divided into four study groups with regard to the donor and recipient age (donor age ≤ 50 years and recipient age ≤ 60 years: D^Y^/R^Y^, *n* = 91; donor age > 50 years and recipient age ≤ 60 years: D^O^/R^Y^, *n* = 38; donor age ≤ 50 years and recipient age > 60 years: D^Y^/R^O^, *n* = 41; donor age > 50 years and recipient age > 60 years: D^O^/R^O^, *n* = 31). Some data were not available or all donors. In this case altered group sizes are displayed within the corresponding line. Detailed results for post-hoc analysis are displayed in Supplementary Table S1. SD, standard deviation.

**Table 3:** Operative outcome.

	D^Y^/R^Y^	D^O^/R^Y^	D^Y^/R^O^	D^O^/R^O^	*p*-Value
**Outcome Variables**	**(*n* = 91)**	**(*n* = 38)**	**(*n* = 41)**	**(*n* = 31)**	
Total graft ischemic time, min (SD)	228 (55)	208 (45)	218 (48)	199 (37)	0.02
Transport time, min (SD)	162 (55)	142 (42)	151 (46)	134 (42)	0.02
Warm ischemia, min (SD)	66 (15)	66 (11)	67 (13)	65 (16)	0.71
Primary graft dysfunction					
Peak catecholamine					
Dobutamine, µg/kg/min (SD)	4.81 (2.16)	5.45 (2.84)	4.40 (2.29)	3.27 (2.43)	0.15
Epinephrine, µg/kg/min (SD)	0.21 (0.18)	0.27 (0.22)	0.20 (0.17)	0.32 (0.23)	0.05
Norepinephrine, µg/kg/min (SD)	0.35 (0.25)	0.37 (0.26)	0.35 (0.34)	0.37 (0.31)	0.94
va-ECMO, *n* (%)	27 (29.7)	10 (26.3)	16 (39.0)	9 (29.0)	0.64
Support duration, d (SD)	9.4 (9.3)	5.7 (5.1)	6.7 (3.6)	9.9 (4.9)	0.34
Deceased on support, *n* (%)	6/26 (23.1)	4/10 (40.0)	2/16 (12.5)	2/8 (25.0)	0.46
Postoperative morbidity					
Infective complications, *n* (%)	19/88 (21.6)	10/36 (27.8)	8/40 (20.0)	12/30 (40.0)	0.21
Acute graft rejection, *n* (%)	7/87 (8.0)	2/36 (5.6)	3/40 (7.5)	2/30 (6.7)	1.00
Hemodialysis on ICU, *n* (%)	43/89 (48.3)	23/37 (62.2)	25/40 (62.5)	19/30 (63.3)	0.27
Neurological complications, *n* (%)	17/88 (19.3)	5/36 (13.9)	7/40 (17.5)	8/30 (26.7)	0.63
Re-thoracotomy, *n* (%)	25/88 (28.4)	12/37 (32.4)	13/40 (32.5)	9/31 (29.0)	0.93
Postoperative hospital stay, d (SD)	42 (28)	41 (24)	51 (39)	54 (52)	0.68
Postoperative ICU/IMC stay, d (SD)	23 (27)	20 (20)	27 (31)	30 (31)	0.20
Mechanical ventilation, h (SD)	145 (197)	109 (141)	197 (210)	183 (232)	0.29
Blood transfusion					
Packed red blood cells, mL (SD)	3716 (5321)	3085 (3186)	3309 (2704)	4646 (5572)	0.70
Fresh frozen plasma, mL (SD)	5646 (8252)	3909 (3179)	6679 (5497)	8802 (8972)	0.09
Platelets, ml (SD)	1012 (2588)	833 (1198)	1106 (1308)	1775 (2719)	0.06
30-day survival, *n* (%)	85/90 (94.4)	32/38 (84.2)	38/40 (95.0)	25/31 (80.6)	0.05
Cause of death within 30 days					0.48
Graft failure	1 (20.0)	1 (16.7)	0 (0.0)	2 (33.3)	
Sepsis/MODS	2 (40.0)	0 (0.0)	0 (0.0)	2 (33.3)	
Coagulopathy	1 (20.0)	2 (33.3)	1 (50.0)	1 (16.7)	
Cerebral injury	1 (20.0.)	0 (0.0)	0 (0.0)	0 (0.0)	
Visceral ischemia	0 (0.0)	0 (0.0)	1 (50.0)	0 (0.0)	
Other/unknown	0 (0.0)	3 (50.0)	0 (0.0)	1 (16.7)	
1-year survival, *n* (%)	62/72 (86.1)	23/31 (74.2)	26/33 (78.8)	14/25 (56.0)	0.02
Cause of death between 30 days and 1 year					0.52
Graft failure	0 (0.0)	0 (0.0)	2 (40.0)	0 (0.0)	
Sepsis/MODS	2 (40.0)	1 (50.0)	1 (20.0)	3 (60.0)	
Coagulopathy	1 (20.0)	0 (0.0)	0 (0.0)	0 (0.0)	
Cerebral injury	0 (0.0)	0 (0.0)	1 (20.0)	0 (0.0)	
Visceral ischemia	1 (20.0)	0 (0.0)	0 (0.0)	0 (0.0)	
Other/unknown	1 (20.0)	1 (50.0)	1 (20.0)	2 (40.0)	

Operative outcome. Patients were divided into four study groups with regard to the donor and recipient age (donor age ≤ 50 years and recipient age ≤ 60 years: D^Y^/R^Y^, *n* = 91; donor age > 50 years and recipient age ≤ 60 years: D^O^/R^Y^, *n* = 38; donor age ≤ 50 years and recipient age > 60 years: D^Y^/R^O^, *n* = 41; donor age > 50 years and recipient age > 60 years: D^O^/R^O^, *n* = 31). Detailed results for post-hoc analysis are displayed in Supplementary Table S1. ICU, intensive care unit; IMC, intermediate care unit; MODS, multiorgan dysfunction syndrome; SD, standard deviation; va-ECMO, veno-arterial extracorporeal life support.

## Data Availability

The data underlying this article will be shared on reasonable request to the corresponding author.

## Supplementary Materials

The following supporting information can be downloaded at: https://www.mdpi.com/article/10.3390/jcm11040929/s1, Table S1: Results of post-hoc analysis.
