# Supplementary material for: Heart Transplantation of the Elderly—Old Donors for Old Recipients: Can We Still Achieve Acceptable Results?
Source: J Clin Med. 2022 Feb 10;11(4):929. doi: 10.3390/jcm11040929 (PMC8877362; doi:10.3390/jcm11040929)
Supplement: Supplementary file 1 [file jcm-11-00929-s001.zip › jcm-1577509-supplementary.pdf]

**Supplementary Table S1**

| Test parameter | $D^Y/R^Y$ vs $D^O/R^Y$ p-value | $D^Y/R^Y$ vs $D^Y/R^O$ p-value | $D^Y/R^Y$ vs $D^O/R^O$ p-value | $D^O/R^Y$ vs $D^Y/R^O$ p-value | $D^O/R^Y$ vs $D^O/R^O$ p-value | $D^Y/R^O$ vs $D^O/R^O$ p-value |
|---|---|---|---|---|---|---|
| **Recipient Variables** | | | | | | |
| Age | 0.99 | <0.01 | <0.01 | <0.01 | <0.01 | 1.00 |
| High urgency status | 0.56 | 0.26 | <0.01 | 0.82 | 0.03 | 0.05 |
| Mechanical ventilation | 0.52 | 0.27 | 0.20 | 0.10 | 0.06 | 1.00 |
| Hemoglobin | 1.00 | 0.22 | 0.37 | 0.26 | 0.37 | 1.00 |
| **Donor Variables** | | | | | | |
| Age | <0.01 | 1.00 | <0.01 | <0.01 | 1.00 | <0.01 |
| Height | 0.14 | 1.00 | 0.77 | 0.13 | 1.00 | 0.59 |
| Body mass index | 0.20 | 1.00 | 0.26 | 0.05 | 1.00 | 0.06 |
| Resuscitation | 0.02 | 0.03 | 0.48 | <0.01 | 0.28 | 0.02 |
| Hypertension | <0.01 | 0.42 | <0.01 | 0.08 | 0.73 | 0.03 |
| Diabetes | 1.00 | 0.16 | 0.04 | 0.17 | 0.18 | <0.01 |
| Drug abuse | 0.28 | 0.09 | 0.19 | 0.03 | 1.00 | 0.02 |
| Lactate dehydrogenase | 0.38 | 1.00 | 1.00 | 0.08 | 1.00 | 0.24 |
| **Outcome Variables** | | | | | | |
| Total ischemic time | 0.26 | 1.00 | 0.02 | 1.00 | 1.00 | 0.31 |
| Transport time | 0.24 | 1.00 | 0.03 | 1.00 | 1.00 | 0.49 |
| Epinephrine | 1.00 | 1.00 | 0.08 | 1.00 | 1.00 | 0.12 |
| 30-day survival | 0.08 | 1.00 | 0.03 | 0.15 | 0.76 | 0.07 |

| | | | | | | |
|---|---|---|---|---|---|---|
| 1-year survival | 0.16 | 0.40 | <0.01 | 0.77 | 0.17 | 0.09 |

Supplementary Table S1 Results of post-hoc analysis. In case of significant results ($p < 0.05$) of Fisher-Freeman-Halton or Kruskal-Wallis test, additional post-hoc analyses by Fisher's exact test or Bonferroni correction were performed.
